# Analizyng the implementation of the Health Promoting University framework in different contexts: a cluster analysis

**DOI:** 10.1590/0034-7167-2024-0130

**Published:** 2025-12-08

**Authors:** Pol Comellas-Sáenz, Dolors Juvinyà-Canal, Sara Esqué-Boldú

**Affiliations:** I University of Andorra. Sant Julià de Lòra, Principality of Andorra; II University of Girona. Girona, Spain

**Keywords:** Health Promotion, Community Participation, Universities, Hierarchical Cluster Analysis, Document Analysis., Promoção da Saúde, Participação da Comunidade, Universidades, Análise por Conglomerados, Análise Documental., Promoción de la Salud, Participación de la Comunidad, Universidades, Análisis por Conglomerados, Análisis de Documentos.

## Abstract

**Objectives:**

to analyse how 22 universities in Spain have implemented the HPU framework in different contexts.

**Methods:**

firstly, a document analysis was used as a research method to assess the action areas of work to promote HPU framework. Documents were selected from the university’s networks and numerically coded.

**Results:**

a Multiple Correspondence Analysis and a Hierarchical Cluster Analysis identified three types of universities based on the implementation of health promotion areas: “Incipient HPU” that is a heterogeneous group that tend to apply some of the HPU principles by addressing acute actions in time; “Premature HPU” englobe those universities that express the need to incorporate a HPU roadmap but does not express it in practice, and “Mature HPU” that are recognized by university authorities.

**Final Considerations:**

these results show how universities recognize and implement the HPU framework differently even when they are part of the same network.

## RESUMO

**Objetivos:**

analisar como 22 universidades na Espanha implementaram a estrutura da Universidade Promotora da Saúde (UPS) em diferentes contextos.

**Métodos:**

primeiramente, utilizou-se a análise documental como método de pesquisa para avaliar as áreas de atuação para promover a estrutura da UPS. Os documentos das redes universitárias foram selecionados e codificados numericamente.

**Resultados:**

uma Análise de Correspondência Múltipla e uma Análise por Conglomerado Hierárquico identificaram três tipos de universidades com base na implementação de áreas de promoção da saúde: “UPS Incipiente”, que é um grupo heterogêneo que tende a aplicar alguns dos princípios da UPS, abordando ações agudas em tempo hábil; “UPS Prematura”, que abrange as universidades que expressam a necessidade de incorporar um roteiro da UPS, mas não a expressam na prática; e “UPS Madura”, que são reconhecidas pelas autoridades universitárias.

**Considerações Finais:**

esses resultados mostram como as universidades reconhecem e implementam a estrutura da UPS de forma diferente, mesmo quando fazem parte da mesma rede.

## RESUMEN

**Objetivos:**

analizar cómo 22 universidades en España implementaron la estructura de la Universidad Promotora de la Salud (UPS) en distintos contextos.

**Métodos:**

en primer lugar, se utilizó el análisis documental como método de investigación para evaluar las áreas de actuación orientadas a promover la estructura de la UPS. Los documentos de las redes universitarias fueron seleccionados y codificados numéricamente.

**Resultados:**

un Análisis de Correspondencias Múltiples y un Análisis de Conglomerados Jerárquicos identificaron tres tipos de universidades en función del grado de implementación de las áreas de promoción de la salud: “UPS incipiente”, grupo heterogéneo que tiende a aplicar algunos de los principios de la UPS mediante acciones puntuales y oportunas; “UPS prematura”, que abarca universidades que manifiestan la intención de incorporar una hoja de ruta hacia la UPS, pero sin concretarla en la práctica; y “UPS madura”, reconocidas por las autoridades universitarias por su consolidación en el modelo.

**Consideraciones finales:**

estos resultados evidencian cómo las universidades reconocen e implementan la estructura de la UPS de manera diversa, incluso cuando forman parte de una misma red.

## INTRODUCTION

Nowadays, the university as a formal institution committed to education play a major role in defining people’s health. Many universities around the globe are adopting new perspectives beyond the classical view for the general diffusion of knowledge^(1)^such as the Health Promoting University (HPU) initiative to health^(2)^. By assuming this approach, universities escaped from the outmoded image as elite institutions for a minority and works to create a supportive environment that promotes the well-being of the entire community^(3)^. The adoption of the setting approach to health promotion involves a change of paradigm, moving from individual-based to community-based intervention. Likewise, the use of this approach implies recognizing the impact of structural and organizational determinants of health rather than individual risk factors^(4)^.

In this scenario, a HPU aims to integrate a commitment to health in all university structures, encouraging participation as an active health promotion strategy and to create an environment that enhance healthy policies and boosts productivity, as well as global well-being and joint action to address shared public health challenges.

### Setting-based Health Promotion

The World Health Organization^(5)^ defines a setting as “the place or social context in which people engage in daily activities in which environmental, organizational, and personal factors interact to affect health and wellbeing”. The settings approach to health promotion moves beyond the mechanistic view and considers the multiple components that make up a whole system to integrate these elements to promote health^(6)^. Thus, the settings are seen as tangible resources to invest for health at a local and international level.

In the last twenty years, a multitude of projects have been developed under the paradigm of healthy settings, from the approach of analyzing work environments^(7)^, to the promotion of hospitals^(8)^, the schools^(9)^, prisons^(10)^ , markets^(11)^ or islands^(12)^.

The setting-based approach in health promotion is based on an ecological understanding of the parts that make up the system. They characterize the setting as having multiple - physical, personal, social, culturaldimensions that can influence the outcomes in health^(13)^. According to the model proposed by Dooris^(14)^ the setting-based approach in health promotion is rooted in three main theoretical frameworks: an ecological model of health^(15)^, a systems perspective^(16)^ and an interest for the whole system organization development^(17)^.

A setting is where people consciously use the environment to create better conditions to live. In this sense of the goal of the setting-approach is to create supportive environments for optimal health. According to Dooris^(18)^ the settings-based approach model to health promotion is underpinned by principles of equity, community, participation, and sustainability. Similarly, in an analysis of the functions of the public health in the new century Lang and Rayner^(19)^ propose an ecological model that address the complexity of the four dimensions of existence. According to them “public health in the 21^st^ century requires policies and actions to engage in all four dimensions to be most effective”. In the same line of argument Kickbusch^(20)^ recognizes the need for a political commitment to improve the health of the entire system to tackle the factors that affect the health of the setting. After a critical revision about the political value of investing in settings for health, Dooris^(14)^ concludes that healthy settings initiatives work towards “the fully developed scenario”.

### A conceptual Framework for Health Promoting Universities

The origins of settings-based health promotion lie within the Ottawa Charter^(21)^, and its global call to re-orientate the practices in public health from the deficit model to focus on the specificities of settings. Following the establishment of the “healthy-settings approach”a movement based in the principles stated in the World Health Organization Health for All Initiatives^(22)^, the Ottawa Charter for Health Promotion^(21)^, and the WHO Healthy Cities Project^(23)^ - in 1995 the University of Central Lancashire established a HPU initiative. In doing so, it became the catalyst for the formation of more local initiatives - collected in the work Health Promoting Universities launched by the WHO^(2)^ - that led to the creation of the European Network of Health Promoting Universities in 1997.

In terms of the functions attributed to health-promoting universities^(24)^ state that the essence of which initiative was developed by a Promote health in the university context should be based on the commitment of the university to incorporate an understanding of sustainable health in all the structures of the organization. This commitment implies: a) to create supportive working, learning, and living environments; b) to promote healthy policies; c) to develop a critical understanding of sustainable health; d) to ensure a healthy physical environment; e) to enable healthy personal and social development; and f) to strength links with the community.

Beyond worrying about improvements in subjective health conditions, the outcomes produced by a HPU should demonstrate the degree to which health has been integrated in the structure, culture, and shape of the organization. Moreover, the higher education’s institutions through their corporate responsibility play a major role in the seek for solutions against sustainability problems that involves different types of settings^(25)^.

An effective Health Promoting University is one that has the capacity to embrace multifaceted frameworks that promotes broader organization change at various levels of the system through the balance of top-down political commitment and bottom-up engagement^(26)^. A forward-looking university would view itself as a social system with inputs, processes, and outputs; would recognize the value of social capital and above all, universities would be respectful of the values of the well-being of its members and will be committed with the resolution of contemporary problems, such climate change and ecology. In this sense, the HPU movement, and the health promotion as an ethos, come together under the challenges presented by the planetary health umbrella^(27)^.

While the key principles and directrices for action about the HPU strategy has been widely and clearly exposed, information about how universities adopt and transform this guidance into pragmatical actions is scarce. To address this issue, the present work aims at describing the characteristics of health promotion initiatives adopted by the universities affiliated to the Xarxa Vives network and seeking to determine the profile of the universities through the implementation of the HPU principles.

## OBJECTIVES

To describe the characteristics of health promotion initiatives adopted by the universities affiliated to the Xarxa Vives network and seeking to determine the profile of the universities through the implementation of the HPU principles.

## METHODS

### Study area and participants

A cross-sectional study was conducted using a Multiple Correspondence Analysis (MCA), followed by a Hierarchical Cluster Analysis (HCA) across twenty-two universities belonging to the XVU network, a non-profit institution that represent and coordinates the joint action of member universities. The purpose of the XVU is to promote relations between the university institutions of Catalonia, the Autonomous Community of Valencia, the Balearic Islands, Northern Catalonia territories, the Principality of Andorra and the Italian island of Sardinia, and also from other territories with common geographical, historical, cultural and linguistic ties, in order to create a university space that allows teaching, research and cultural activities to be coordinated as well as to promote the use and normalization of the Catalan language.

### Study design and instruments

The research comprised two phases. In phase 1 to explore the implementation of programs for promoting health within the university context an analysis of the documents shared publicly was organized among universities belonging to Xarxa Vives University network in different countries. As part of a larger study, the 22 universities affiliated to the network were contacted and invited to participate through the dissemination of internal communiqués. For the first review, all internal university communications and documents were imported in a database guided by a protocol based on the recommendations for document analysis technique^(28)^. After analyzing the characteristics of the actions for promoting healthy habits contained in the notes, using the content analysis tool, in phase two the different items of work were classified into theoretical variables. For further, all the items of work were contrasted and refined using the headings of work proposed by Dooris et al.^(29)^ to support healthy universities in England, and the areas of action assessed by Suárez-Reyes et al.^(30)^ when comparing different HPU initiatives worldwide. The analytical design covered the following aspects: (1) General information about the university, which included type of funding, location, university size, etc.; (2) Information about the presence of a HPU initiative, which inquired about the membership of a HPU network and the institutional agent for the implementation of the health promotion initiatives; (3) Priority areas of action or work dimensions, which explored the broader areas of work; (4) Items of work or specific actions, which examined the activities and the actions taken for the university in order to promote a healthy habit; (5) Evaluation, which concerned any evaluation process of the initiative. All these dimensions were coded using three numerical options (yes/no/maybe) based on the implementation of the programs.

Public such as reports, monographs, presentations, podcast among others were extracted from the web domains of each of the twenty-two universities that belongs to the XVU network separately. This channel was chosen as the primary data source over other methodological procedures due to the higher content related to the institutional commitment to health promotion practices.

The data were retrieved directly from the universities webpages during a one-month period, in January 2024. Data extraction was done separately by all authors.

### Study design and data sources

This was a cross sectional study. We used publicly available data on multiple HPU dimensions extracted from the university’s webpages. Qualitative data analysis software (Atlas ti) was used to organize and code the imported documents. The list of codes resulting from this process served as a framework for the subsequent coding of transcripts. Where available, we used the most recent data for each data source.

No ethics review was sought because the study only explored the publicly available data on social media and did not conduct any experiments on human participants. However, the Ethics Committee of the University of Andorra approved the study protocol on May 09, 2023.

### Data collection, and filtering

Between January 1, 2024 and February 1, 204, we archived all the documents, posts and associated metadata published by each university with no restriction. As a second step, we filtered and classified the collected information to retain those related to the health promotion practices. To this end, we filtered collected activities based on the topics present in the communications from the webpages. To facilitate this, we established a list of thematic domains related to health promotion activities. To develop the list of HPU related activities, we used relevant literature^(30)^ based on previous studies. Included information were required to be explicitly related to health promotion.

### Statistical Analysis

To derive information from the coded documents, descriptive statistics (frequencies and percentages) were computed regarding the dimensions of work addressed by the universities. Later on, the wide dataset was utilized to study the relationships between the university characteristics and the implementation of health promotion programs using chi-square tests, whereas the long dataset regarding the items of work was used to group universities using a Multiple Correspondence Analysis, followed by a Hierarchical Cluster Analysis.

### Multiple Correspondence Analysis

Considering descriptive results and the frequencies of the occurrence of the different HPU dimensions, we selected ten variables (physical activity, diet, emotional health, sexual health, health at work, transport, tobacco consumption, sustainability, and institutional collaboration) to be analyzed using MCA. These were all binomial variables.

MCA detects underlying structures in a set of nominal or categorical data by graphically representing data as points in a low-dimensional Euclidean space. This graphical display allows to investigate the pattern of relationships of the variables. Thus, the distance between universities and areas of action was determined by considering the attributes they shared. All these internal structures can be assessed in a factor map.

### Hierarchical Cluster Analysis

Based on the dimensions resulted on the MCA, a HCA was performed using Ward’s minimum variance method^(31)^ to generate homogenous clusters of universities sharing similar health promotion profiles. After visualizing the dendrogram generated by calculating the distance between each pair of universities, Chi-square test was used to test differences between clusters regarding sociodemographic characteristics (Table 1) and items of work. As recommended by Husson et al.^(32)^, we included only the eigenvalues that represent a summarized variance of 80% of the overall variance only.

**Table 1:** Characteristics of the universities participating (N=22)

Variable	n	%
*Country*		
Andorra	1	(4.54%)
Spain • Catalunya • Balearic Islands • Valencia	12 1 6	(54.54%) (4.54%) (27.27%)
France	1	(4.54%)
Italy	1	(4.54%)
*Type* Public Private	16 6	(72.72%) (27.27%)
*University size* Small <10.000 Medium >10.000 <20.000 Big >20.000	3 10 9	(13.63%) (45.45%) (40.90%)
*Ranking classification* ^a^ Low >1000 Medium >500 <1000 High <500 No information	2 13 3 4	(9.09%) (59.09%) (13.63%) (18.18%)
*Fees in euro/ first year European student* <1500 >3000 <5000 < 5000	15 3 4	(68.18%) (9,09%) (18,18%)
*HPU Network participation* Yes No	19 3	(86.36%) (13.63%)

*The values represent the number of universities (percentages); aTimes Higher Education ranking 2023.*

As the study did not involve human subjects, obtaining informed consent was not applicable.

## RESULTS

From the Xarxa Vives University network we collected information from the 22 universities that are members of it regarding different topics on health promotion. From a list of eight original areas of action, the items of work most often addressed by the universities responsible were: physical activity, healthy diet interventions, emotional health, and health at workplace. Other priority areas identified through the document analysis included transport, tobacco policies, drug abuse campaigns and sexual health.

Table 2 shows examples of concrete activities that universities address to implement the areas of action.

**Table 2:** Specific examples of activities to develop the Health Promoting University items of work

Physical Activity	Capsules containing information; healthy routes on campus; use of the university’s sports facilities; Student Health survey; free activities for students and staff; practical guide with exercises to do at home.
Diet	Decalogue for a healthy lunch box; advice on diet drawn up by experts on dietetics; workshops; agreements with catering services to offer healthy options
Emotional health	Capsules with tips on mental health and meditation; psychological services providing counselling; workshops for students and staff.
Sexual Health	Capsules with tips on sexual health; counselling services on sexual health; awareness campaigns using infographics on campus facilities; sexual health and courses of STD
Drug Abuse	Awareness campaigns; advisory service and counselling; non-smoking day project; workshops for detection and prevention of the addictive behaviour
Health at workplace	Health screening for students and staff; advice and exercises for maintaining a healthy body position at workplace; physiotherapeutic services; medical services providing counselling
Tobacco policy	Celebration of the non-smoking day on campus; smoking free spaces on campus specific areas
Transport	Promotion of bicycle transportation; provision of bicycles and scooters parking spaces; carsharing apps for university members; provision of showers on campus

*The actions illustrated in the different categories are practical examples removed using the document analysis technique.*

### Is there a common typology of Health Promoting University within the XVU network?

**Figure 1:**
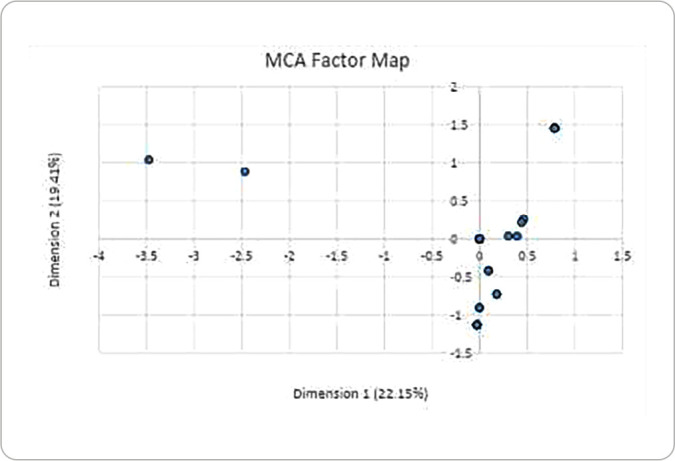
Factor map

Figure 1 present the 2D solution of the 22 universities that integrates the XVU network for the different areas of action that has been identified previously in the literature. Overall, ten dependent variables with three answer categories were included. While MCA technique allowed for the reduction of data by grouping it in a common space or axis, in a subsequent step HCA was performed to cluster universities according to the HPU key principles. Both methods are analytical tools that were purposive used to explore and underly similar patterns or clusters within the universities. All the decisions taken in the moderation of theoretical configuration were made after discussion within the research team.

The overall variance of the data was 41.57% and could be displayed on two axes: the 22.156% (0.179/1.3) of total inertia accumulated for Dimension 1 and the 19.415% (0.647/1.3) for Dimension second. The MCA model total inertia was 1.3.

The contribution of the variables for the first two dimensions are presented in Table 3. When the values are close to 1, the greater is the contribution of the variable to the dimension definition. On the contrary, values close to 0 indicate little discriminating capacity, and therefore, a lower explanatory weight of the variable to the model heterogeneity. In order to gain a greater insight of the relationship between variables, all recorded values higher than the inertia score have been considered significant in the preparation and explanation of the factor map.

**Table 3:** Variables contribution

Variables	Dimension 1 (axis 1/x-axis)		Dimension 2 (axis 2/y-axis)		Mean
	Discrimination	Contribution (%)	Discrimination	Contribution (%)	
Physical Activity	0.584	7.826	0.496	7.534	0.540
Diet	0.589	7.893	0.686a	10.420	0.637
Drug Abuse	0.955^a^	12.798	0.822^a^	12.486	0.889
Emotional Health	0.927^a^	12.422	0.618	9.387	0.772
Sexual Health	0.955^a^	12.798	0.822^a^	12.486	0.889
Transport	0.956^a^	12.811	0.772^a^	11.727	0.864
Tobacco policy	0.955^a^	12.798	0.822^a^	12.486	0.889
Health at workplace	0.941^a^	12.610	0.577	8.765	0.759
Collaboration	0.261	3.497	0.334	5.073	0.298
Sustainability	0.262	3.511	0.523	7.944	0.393
Ranking	0.077	1.031	0.111	1.686	0.094
Total	7.462	100.000	6.583	100.000	7.024
% of the inertia projected	22.156		19.415		20.786
Inertia	0.739		0.647		

a The values in italics are considered high and significance (above inertia).

We can interpret from the Figure 1, that the first-dimension distinguished items of work that focus on the structural and community-centered health promotion interventions. Then, second dimension groups materialsmore individualistic oriented actions. For instance, the top-left quadrant groups two universities whose initiatives were mentioned in theory but not performed in action; whereas the bottom-right quadrant recognize those universities who may present a steering group to coordinate specific health promotion activities and receive institutional recognition through funding and multisectoral collaboration.

Based on the object scores of MCA, a HCA was performed to group the universities according to how they implement health promotion areas of action. Based on the visual inspection of the dendrogram, a tree diagram describing the steps required by the unique clustering technique to a unique cluster with *n* cases, three to five clusters were retained. Considering the lack of clarity reported by the diagram, and the subjectivity in the interpretation of the results the existence of two models were configured according to the coefficients reported. The comparisons between these clusters for the areas of action of HPU are shown in Table 4. There were significant differences between clusters on all ten components. Furthermore, no significant differences were found for the descriptive variables presented in Table 1 among the three groups.

**Table 4:** Comparisons among clusters showing different Health Promoting University initiatives

	Total N=22	Cluster 1 N=15	Cluster 2 N=2	Cluster 3 N=5	*p* value
Physical Activity	14 (63.63%)	8 (53.33%)	1 (100%)	5 (100%)	0.157
Diet	12 (54.54%)	6 (40.00%)	1 (77.77%)	5 (100%)	0.067
Drug Abuse	5 (22.72%)	0 (0%)	0 (0%)	5 (100%)	<.001
Emotional health	8 (%)	3 (8.66%)	0 (0%)	5 (100%)	<.001
Sexual health	5 (22.72%)	0 (0%)	0 (0%)	5 (100%)	<.001
Transport	7 (31.81%)	2 (13.33%)	0 (0%)	5 (100%)	<.001
Tobacco policy	5 (22.72%)	0 (0%)	0 (0%)	5 (100%)	<.001
Health at workplace	10 (45%)	5 (33.33%)	0 (0%)	5 (100%)	0.021
Collaboration	10 (45%)	5 (33.33%)	0 (0%)	5 (100%)	<.001
Sustainability	9 (40.90%)	4 (26.66%)	0 (0%)	5 (100%)	<.001

*The values represent the number of universities (percentage) in relation to each cluster. p value; significance.*

After the evaluation of the results derived from the documentary analysis, three clusters have been generated. Cluster 1 is informed by 15 universities (68.18% from the 22 universities that integrates the XVU network); Cluster 2 by 2 universities (9.09% from the 22 universities that integrates the total network); and Cluster 3 is integrated by 5 institutions (22.72%). Based on these comparisons, the clusters can be characterized as follows:

Cluster 1 is the largest and most heterogeneous grouping of the proposed model and is made up of 15 universities of different types, sizes, and international recognition. The institutions included in this cluster present concrete actions to encourage the acquisition of healthy habits, mainly through the institutional collaboration to offer sport services or emotional counselling. In general terms, a large part of the stated initiatives is developed through concrete actions in time, most of them without a follow-up control. Looking forward the multiplicity of action oriented to promote health inside of the community, the initiatives included in this cluster could be labelled as” incipient HPU”.

Cluster 2 is outlined by two private universities with variable size depending on the institution. The first ones were coded as a small university (n<1500 students) and the second one as medium (n>1500). Both higher education institutions publicly express the need to incorporate a strategic health promotion plan into the university’s road map. However, this willingness is not shown in practice. Both universities belong to a HPU network.

Respecting the will to develop a plan on HPU initiative, the universities highlighted by this cluster can be labelled as “Premature Health Promoting Universities”.

Unlike the two clusters mentioned above, Cluster 3 is informed by five medium-large public universities with international recognition that place them in leading positions within the Iberian Peninsula. All five institutions present a strategic framework for action informed by a steering group, most of them recognized as “healthy university plan” or “health office”. The actions implemented are recognized by university agents and are supported in it’s majority by economical resources and institutional bonds with other departmental sections, from the university itself or external to it. All five universities belong to a broader HPU network at the national level. Considering the solid structuring of the institutional HPU key elements, the initiatives included in this cluster can be labeled as “Mature Health Promoting Universities”.

## DISCUSSION

We have argued that HPU movement, as part of a larger multidisciplinary strategy in health promotion, is an ambiguous and broad concept consisting of many aspects that need to be addressed before it becomes a reality. In order to contribute to the better understanding of the opportunities in some universities of the Mediterranean coast today, we examined publications to obtain an empirical approach on the initiatives and opportunities mobilized by some universities to become a Health Promoting setting. The results we present here are exploratory and specific from a local region. However, our findings help us better understand how the HPU movement is being implemented in some national universities and, subsequently, what are the practical opportunities to prompt new initiatives inside of the XVU network in the near future.

### Actions areas *or what we do*

Regardless of the type of university, the number of students it hosts or the membership to a HPU network, the more often addressed interventions seeks to promote physical activity and emotional counselling as the cornerstones of a healthy lifestyle. This result is not surprisingly in the sense that mental health and sedentary behaviors are two of the main worries concerning the health status of the student college community^(33)^. In a cross-sectional study supported by international collaboration between 19 independent institutions in 8 different countries, the authors revealed that almost the 35% of the students met at least one mental health diagnostic criterion^(34)^. Additionally, there is evidence that most of the student population spends too many hours per day sitting^(35)^, what has been related to one’s health damaging outcomes as obesity or even death^(36)^.

The results of the already available documents reviewed and coded above support the hypothesis that university students are seen as some kind of vulnerable population suffering from different mental problems including, anxiety, depression, and stress. Early drug initiation increases de risk of drug progression at different stages. Numerous retrospective studies have found a positive association between alcohol use and later use of marijuana and cocaine or other illicit drugs^(37,38)^. Regarding this pattern of drug abuse consumption, some authors have considered tobacco and alcohol as “gateway” substances^(39,40)^ which challenges universities to generate new interventions programs to prevent, control and promote healthy decision making primarily with respect to the students group.

Our findings also reveal a three-model theoretical structure of the XVU universities relating to the implementation of the HPU concept. Clearly, physical activity and psychological counselling are seen for institutions as major topics to address. However, while the evidence on HPU studies suggest a predominance for single health topic interventions^(41)^only a small number of universities could be found that describe the implementation of programs focusing on the entire community (cluster three). These results found in practice are aligned with other theoretically reported evidence^(42)^.

The health promotion initiatives were consistent with the verbatim stated by the professionals when compared to the advantages and limitations at each university. Higher education’s systems implement health promotion practices from a reductionist perspective. Only a small number of universities could be found that describe the implementation of programmes adopting a whole systems approach. This analytical review included all the universities that integrates the XVU network, mainly in the region of Catalonia (Spain), but also in Andorra, South of France, and one Italian university. Similar results have been found when analyzing the implementation of single issues in health in other international HPU member networks^(30)^.

### Action areas or *what’s next*

Various guidelines in health promotion have emphasized the importance in adopting a setting-based approach to health promotion when addressing interventions to health in all campus aspects^(2,43)^. However, the reviews made up to date aligning with the HPU concept reveals a different speed in the adoption of the HPU principles^(42,44,45)^.

One of the principles goals of the Okanagan Charter for Health Promotion is to provide institutions a common framework to become a health and wellbeing promoting campus^(43)^. The results of the current study have important implications regarding this goal within the universities members of the local networks in the Mediterranean Coast. Efforts to establish institutional collaboration toward universities sectorial practices are an important strategy to guarantee the effectiveness of the actions undertaken in the context of health promotion^(46)^. To accomplish this, the contribution of all sectors is required beyond the idea that an HPU initiative only seeks to change people health. Rather, actions areas such as sustainability or transport are essential issues to consider within such a HPU project. In this sense, the model proposed by Wamslaer and colleagues^(47)^ can provide a good starting point from which to start seeing topics like climate change as human relationships problems rather than merely environmental things. Recent research examining health-promoting and sustainable behavior in university students^(48)^ will facilitate the development of new interventions in this setting, which should be an important target for health promoting universities enterprises.

To the best of our knowledge, this is the first study to assess the programs and items of work in health promotion among the universities that integrates the XVU network. In addition to local network membership, the 72% of the universities are part of the national HPU network, *Red Española de Universidades Promotoras de la Salud* (REUS), committed to embed health within the university community based on the institutional support of the public sectors and the active initiative together with local and national governments^(49)^. However, the health status impact of being part of an HPU network has not yet been empirically evaluated.

The results depicted from this review should be interpreted within the HPU framework as a timely catalyst for future research. At the time of the literature and actions codification, only 2 out of 22 universities were implementing a Healthy University project, which represents less than a quarter of the total universities members. Further research is required to assure the future of the HPU setting approach in the Xarxa Vives University network and across borders.

### Study limitations

This study has several limitations. Firstly, regardless the depth in which the documents of all the participating universities have been analyzed, the statistical methodology used represents a first structured approach to the analysis of the XVU universities. Reliability might have been enhanced if more than one reviewer had been involved in screening and coding data. Secondly, this global did not extend beyond the institutions who integrate the XVU network. Further analysis of the practical guidelines in other HPU networks is needed. Thirdly, notwithstanding active consideration on the cluster analysis done, the inclusion of other criteria in the reasoning, such as health indicators or institutional funding, would might provide a different bunch. Including ecumenical research tool as questionnaires or other data collection techniques would have allowed us to deepen our understanding of the perceptions of the different actors involved in the university setting.

Finally, we did not set out to evaluate the implementation of the interventions reported from the webpages, nor the validity of the health promotion actions.

### Contributions to the nursing area

Firstly, since the 1980s, when health promotion began to diverge from the traditional biomedical model, the conceptual foundations of public health practice have at times become detached from their original purpose. In this regard, understanding the university context as an agent in itself necessitates examining how the various professionals who interact within it can contribute to improving the quality of life and well-being of the entire community.

In this context, it becomes imperative to rebuild professional bridges between organizations to ensure the comprehensiveness of proposed initiatives beyond traditional academic obligations, and thus continue the pursuit of an ideal that is not predetermined, but rather shaped by the demands of a given historical moment.

## CONCLUSIONS

This review reveals that the implementation of the HPU key principles is scarce. Notwithstanding specific actions oriented to health promotion could be identified at the university setting, the scope of these guidelines and practical basis was predominantly oriented toward a selected number of individual behavioral interventions using an individualistic approach. While the gold standard, as have been noticed elsewhere^(50)^ advocates for a holistic and comprehensible approach of the parts that make up the system, what has been named the whole system approach, the implementation of the leading concepts is still far from be reached.

From the pragmatical perspective, the analysis made reinforces the importance of university commitment to health through the creation of a steering group and institutional bonds inside and outside the university setting to provide enabling learning environments that are positive to induce a change in the community habits, which will benefit from both top-down and bottom-up paths. In this sense, a germinal priority is to establish a common road map in between the institutions which participate from the network resources and to align the common synergies to the needs identified by each university. The current results can guide the development of a framework for joint reflection towards the implementation of the HPU initiatives, which we hope, will lead institutions to their way of becoming healthier places to study, play, work, and love, or what it takes.

## Data Availability

The research data are available within the article.

## References

[1] Ford M. (2017). The Functions of Higher Education. Am J Econ Sociol.

[2] Tsouros A, Dowding G, Thompson J, Dooris M. (1998). Health Promoting University: Concept, Experience and Framework for Action. World Health Organisation.

[3] Cawood J, Dooris M, Powell S. (2010). Healthy universities: shaping the future. Perspect Public Health.

[4] Dooris M. (2009). Holistic and sustainable health improvement: the contribution of the settings-based approach to health promotion. Perspect Public Health.

[5] Nutbeam D, Muscat DM. (2021). Health Promotion Glossary 2021. Health Promot Int.

[6] Poland B, Krupa G, McCall D. (2009). Settings for health promotion: an analytic framework to guide intervention design and implementation. Health Promot Pract.

[7] The Lancet Public Health (2022). Healthy workplaces for a healthy living. Lancet Public Health.

[8] Whitehead D. (2005). Health promoting hospitals: the role and function of nursing. J Clin Nurs.

[9] Lee A. (2009). Health-promoting schools: evidence for a holistic approach to promoting health and improving health literacy. Appl Health Econ Health Policy.

[10] Woodall J, Dixey R, South J. (2014). Control and choice in English prisons: developing health-promoting prisons. Health Promot Int.

[11] Holmes C, World Health Organization (2004). Regional Office for the Western Pacific. Healthy marketplaces in the Western Pacific: guiding future action: applying a settings approach to the promotion of health in marketplaces.

[12] Galea G, Powis B, Tamplin SA. (2000). Healthy islands in the Western Pacific - International settings development. Health Promot Int.

[13] Stokols D. (1996). Translating social ecological theory into guidelines for community health promotion. Am J Health Promot.

[14] Dooris M. (2004). Joining up settings for health: a valuable investment for strategic partnerships?. Crit Public Health.

[15] Whitelaw S, Baxendale A, Bryce C, MacHardy L, Young I, Witney E. (2001). ‘Settings’ based health promotion: a review. Health Promot Int.

[16] Paton K, Sengupta S, Hassan L. (2005). Settings, systems and organization development: the Healthy Living and Working Model. Health Promot Int.

[17] Grossman R, Scala K. (1993). Health Promotion and Organisational Development: developing dettings for health. WHO Regional Office for Europe;.

[18] Dooris M. (2006). Healthy settings: challenges to generating evidence of effectiveness. Health Promot Int.

[19] Lang T, Rayner G. (2012). Ecological public health: the 21st century’s big idea? an essay by Tim Lang and Geof Rayner. BMJ.

[20] Kickbusch I. (2003). The contribution of the World Health Organization to a new public health and health promotion. Am J Public Health.

[21] World Health Organization (WHO) (1986). Ottawa Charter for Health Promotion. Ottawa.

[22] World Health Organization (WHO) (1980). Targets for Health for All. WHO Regional Office for Europe.

[23] Tsouros AD. (1995). The WHO Healthy Cities Project: state of the art and future plans. Health Promot Int.

[24] Booth B, Ritchie B., Tsouros A, Dowding G, Thompson J, Dooris M (1998). Health Promoting Universities: Concept, Experiences and Framework for action.

[25] Orme J, Dooris M. (2010). Integrating health and sustainability: the higher education sector as a timely catalyst. Health Educ Res.

[26] Dooris M. (2022). In: Handbook of Settings-Based Health Promotion.

[27] Hancock T, IUHPE’s Global Working Group on Waiora Planetary Health (2021). Towards healthy One Planet cities and communities: planetary health promotion at the local level. Health Promot Int.

[28] Bowen GA. (2009). Document analysis as a qualitative research method. Qual Res J.

[29] Dooris M, Farrier A, Doherty S, Holt M, Monk R, Powell S. (2018). The UK Healthy Universities Self-Review Tool: whole system impact. Health Promot Int.

[30] Suárez-Reyes M, Muñoz-Serrano M, Van den Broucke S. (2019). How do universities implement the Health Promoting University concept?. Health Promot Int.

[31] Ward JH. (1963). Hierarchical Grouping to Optimize an Objective Function. J Am Stat Assoc.

[32] Husson F, Lê S, Pagès J. (2017). Exploratory Multivariate Analysis by Example Using R: exploratory multivariate analysis by example Using R.

[33] World Health Organization (WHO) (2017). Depression and Other Common Mental Disorders.

[34] Auerbach RP, Alonso J, Axinn WG, Cuijpers P, Ebert DD, Green JG (2016). Mental disorders among college students in the World Health Organization World Mental Health Surveys. Psychol Med.

[35] Park JH, Moon JH, Kim HJ, Kong MH, Oh YH. (2020). Sedentary Lifestyle: overview of updated evidence of potential health risks. Korean J Fam Med.

[36] Chau JY, Grunseit AC, Chey T, Stamatakis E, Brown WJ, Matthews CE (2013). Daily sitting time and all-cause mortality: a meta-analysis. PLoS One.

[37] Busto Miramontes A, Moure-Rodríguez L, Díaz-Geada A (2019). Heavy drinking and non-medical use of prescription drugs among university students: a 9-year follow-up. Int J Environ Res Public Health.

[38] López Steinmetz LC, Leyes CA, Fong SB, Godoy JC. (2023). Alcohol consumption, alcohol expectancies, and drinking contexts in young Argentinean college students before and during the COVID-19 pandemic: a one-year follow-up study. Am J Drug Alcohol Abuse.

[39] Nkansah-Amankra S, Minelli M. (2016). “Gateway hypothesis” and early drug use: additional findings from tracking a population-based sample of adolescents to adulthood. Prev Med Rep.

[40] Nkansah-Amankra S. (2020). Revisiting the association between “Gateway Hypothesis” of early drug use and drug use progression: a cohort analysis of peer influences on drug use progression among a population cohort. Subst Use Misuse.

[41] Dooris M, Wills J, Newton J. (2014). Theorizing healthy settings: a critical discussion with reference to Healthy Universities. Scand J Public Health.

[42] Suárez-Reyes M, Van den Broucke S. (2016). Implementing the Health Promoting University approach in culturally different contexts: a systematic review. Glob Health Promot.

[43] International Conference on Health Promoting Universities & Colleges (2015). In: Okanagan Charter : an international charter for health promoting universities & colleges.

[44] Reis M, Ramiro L, Gómez-Baya D, Matos MG. (2018). The Promotion of Healthy Universities: A Systematic Review. CPQ Women and Child Health.

[45] Sweeting H, Thomson H, Wells V, Flowers P. (2023). Evolution of ‘whole institution’ approaches to improving health in tertiary education settings: a critical scoping review. Res Pap Educ.

[46] Dooris M, Powell S, Parkin D, Farrier A. (2021). Health promoting universities: effective leadership for health, well-being and sustainability. Health Educ.

[47] Wamsler C, Osberg G, Osika W, Herndersson H, Mundaca L. (2021). Linking internal and external transformation for sustainability and climate action: towards a new research and policy agenda. Glob Environ Change.

[48] Weber A, Kroiss K, Reismann L, Jansen P, Hirschfelder G, Sedlmeier AM (2023). Health-Promoting and sustainable behavior in university students in Germany: a cross-sectional study. Int J Environ Res Public Health.

[49] Martínez-Sánchez JM, Balaguer A. (2016). Healthy university: a health promotion strategy and health for all policies for the creation of a healthy workplace. Arch Prev Riesgos Labor.

[50] Thompson SR, Watson MC, Tilford S. (2018). The Ottawa Charter 30 years on: still an important standard for health promotion. Int J Health Promot Educ.

